# The *Thiamin-Requiring 3* Mutation of Arabidopsis *5-Deoxyxylulose-Phosphate Synthase 1* Highlights How the Thiamin Economy Impacts the Methylerythritol 4-Phosphate Pathway

**DOI:** 10.3389/fpls.2021.721391

**Published:** 2021-08-06

**Authors:** Jaya Joshi, Manaki Mimura, Masaharu Suzuki, Shan Wu, Jesse F. Gregory, Andrew D. Hanson, Donald R. McCarty

**Affiliations:** ^1^Department of Horticultural Sciences, University of Florida, Gainesville, FL, United States; ^2^Plant Cytogenetics, Department of Gene Function and Phenomics, National Institute of Genetics, Mishima, Japan; ^3^Department Food Science and Human Nutrition, University of Florida, Gainesville, FL, United States

**Keywords:** thiamin, methylerythritol 4-phosphate pathway, 5-deoxyxylulose-phosphate synthase, thiamin requiring, retrograde signaling, biotic stress response

## Abstract

The thiamin-requiring mutants of Arabidopsis have a storied history as a foundational model for biochemical genetics in plants and have illuminated the central role of thiamin in metabolism. Recent integrative genetic and biochemical analyses of thiamin biosynthesis and utilization imply that leaf metabolism normally operates close to thiamin-limiting conditions. Thus, the mechanisms that allocate thiamin-diphosphate (ThDP) cofactor among the diverse thiamin-dependent enzymes localized in plastids, mitochondria, peroxisomes, and the cytosol comprise an intricate thiamin economy. Here, we show that the classical *thiamin-requiring 3* (*th3*) mutant is a point mutation in plastid localized *5-deoxyxylulose synthase 1* (*DXS1*), a key regulated enzyme in the methylerythritol 4-phosphate (MEP) isoprene biosynthesis pathway. Substitution of a lysine for a highly conserved glutamate residue (E323) located at the subunit interface of the homodimeric enzyme conditions a hypomorphic phenotype that can be rescued by supplying low concentrations of thiamin in the medium. Analysis of leaf thiamin vitamers showed that supplementing the medium with thiamin increased total ThDP content in both wild type and *th3* mutant plants, supporting a hypothesis that the mutant DXS1 enzyme has a reduced affinity for the ThDP cofactor. An unexpected upregulation of a suite of biotic-stress-response genes associated with accumulation of downstream MEP intermediate MEcPP suggests that *th3* causes mis-regulation of DXS1 activity in thiamin-supplemented plants. Overall, these results highlight that the central role of ThDP availability in regulation of DXS1 activity and flux through the MEP pathway.

## Introduction

Thiamin is an essential cofactor for central metabolism of all living organisms. Bacteria, fungi, and plants are capable of synthesizing thiamin (Jurgenson et al., 2009). The classical thiamin-requiring mutants of Arabidopsis and other plants have been extensively studied as a model for biochemical genetics of plants (Langridge, 1955, 1965; Li and Rédei, 1969; Koornneef, 1981). Nevertheless, several processes in thiamin biosynthesis and salvage in plants have not yet been connected with corresponding genes (Gerdes et al., 2012) indicating there is more that can be learned from molecular analysis of thiamin-requiring mutants. We recently determined that the classical *thiamin-requiring 2* (*th2*) gene described by (Langridge, 1955, 1965) encodes the orphan thiamin biosynthesis pathway enzyme thiamin monophosphatase (Mimura et al., 2016). Another uncharacterized classical thiamin-requiring mutant, *thiamin-requiring 3* (*th3*), is identified by a single mutant allele described by Koornneef (1981).

Several modes for regulation of the thiamin biosynthesis have been identified. These include a conserved riboswitch mechanism for feedback control of the pyrimidine branch of the biosynthesis by thiamin-diphosphate (ThDP; Croft et al., 2007; Wachter et al., 2007; Bocobza et al., 2013), diurnal and circadian regulation of thiamin biosynthesis (Rosado-Souza et al., 2019; Noordally et al., 2020), and protein turnover (Li et al., 2017). Although the quantity of thiamin cofactor required for plant growth and development is small, on a molar basis thiamin biosynthesis is energetically expensive due to costs of enzyme synthesis and turnover (Hanson et al., 2016). The limiting reaction in synthesis of the thiazole moiety, THI4, is catalyzed by a single-turnover suicide protein (Chatterjee et al., 2011; Joshi et al., 2020). Consequently, a molecule of THI4 protein is consumed for every molecule of thiamin synthesized. This costly reaction can account for the high rates of THI4 protein turnover observed in barley and Arabidopsis leaves (Nelson et al., 2014; Li et al., 2017). Similarly, a key enzyme in the pyrimidine branch of the thiamin biosynthetic pathway, THIC (Raschke et al., 2007; Kong et al., 2008), catalyzes only a few catalytic turnovers per molecule of enzyme (Hanson et al., 2016). Thiamin synthesis has been broadly implicated in plant stress tolerance (Sayed and Gadallah, 2002; Tunc-Ozdemir et al., 2009; Hanson et al., 2016) and disease resistance (Ahn et al., 2005; Wang et al., 2006).

The complexity of thiamin-dependent metabolism in plants implies an intricate thiamin economy for efficient allocation of an apparently limiting cofactor resource (Gerdes et al., 2012; Colinas and Fitzpatrick, 2015; Hanson et al., 2016; Fitzpatrick and Chapman, 2020). In leaf tissue, ThDP content is comparable to the estimated total number of enzyme active sites indicating that most thiamin-dependent enzymes are typically only just fully activated (Hanson et al., 2016; Joshi et al., 2019). Cofactor allocation is further complicated by the localization of thiamin-requiring enzymes in at least four cell compartments, plastids, mitochondria, peroxisome, and cytosol. Moreover, the thiamin biosynthetic and salvage pathways are distributed among multiple compartments (Gerdes et al., 2012; Colinas and Fitzpatrick, 2015). Dynamic changes in ThDP content also occur in the nucleus (Noordally et al., 2020). The mechanisms that control transport of thiamin and its vitamers between cells (Woodward et al., 2010; Guan et al., 2014; Martinis et al., 2016) and among cellular compartments (Beaudoin et al., 2018; Noordally et al., 2020) to facilitate allocation among enzymes remain poorly understood. The complex phenotype induced by overexpression of transketolase apo-protein in tobacco leaves indicates that comparatively mild perturbations of the thiamin economy can have unexpected effects on metabolism, development, and thiamin biosynthesis (Khozaei et al., 2015).

The thiamin-requiring enzyme deoxyxylulose 5-phosphate synthase 1 (DXS1) catalyzes the first committed step in the methylerythritol 4-phosphate (MEP) pathway for isoprene biosynthesis (Zhao et al., 2013) localized in the chloroplast (Banerjee et al., 2013; Wright et al., 2014; Pulido et al., 2016). Diurnal regulation of the MEP pathway has been studied extensively in plants (Wright et al., 2014; Pokhilko et al., 2015). DXS1 is regulated in part by protein turnover by the chloroplast CLP pathway where DXS1 is targeted for degradation by interaction with an Hsp100 chaperonin (Pulido et al., 2016). Competitive inhibition of ThDP binding to DXS by MEP pathway end product, Dimethylallyl pyrophosphate (DMAPP; Banerjee et al., 2013; Sharkey et al., 2015) has been proposed as a mechanism for feedback regulation of DXS activity. A downstream intermediate in the MEP pathway, MEcPP, is implicated in retrograde signaling by chloroplasts under stress conditions (Xiao et al., 2012; González-Cabanelas et al., 2015; Benn et al., 2016; Lemos et al., 2016; Bjornson et al., 2017). Accumulation of MEcPP in mutants of the hydroxy-3-methylbut-2-enyl diphosphate synthase (*HDS*) gene in the pathway results in upregulation of a suite of genes implicated in biotic stress responses (Lemos et al., 2016; Bjornson et al., 2017).

Here, we show that the classical thiamin-requiring *th3* mutation of Arabidopsis described by Koornneef (1981) is an allele of *DXS1 [Cloroplastos Alterados 1 (CLA1)]*. While the pale-green leaf phenotype of *th3* is consistent with reduced flux through the MEP pathway, vitamer contents of *th3* mutant plants grown on thiamin-supplemented medium were similar to those of wild type indicating that thiamin metabolism was unaltered. The *th3* mutation substitutes lysine for a highly conserved glutamate residue located at the homodimer interface of DXS1. We suggest that the mutant enzyme has a lower affinity for ThDP causing reduced activation of DXS1 *in vivo* and that activity is restored by elevated ThDP concentration in plants grown on medium supplemented with thiamin. Unexpectedly, *th3* mutant plants exhibit upregulation of a suite of biotic stress-response genes that are also induced by accumulation of the downstream MEP pathway intermediate, MEcPP. We consequently infer that DXS1-th3 has substantial knock-on effects on the whole MEP pathway.

## Materials and Methods

### Plant Materials

Seed of the *th3* stock (CS81) and reference *cla1-1S* DXS1 stock (CS16003) were obtained from the Arabidopsis Biological Resource Center.^1^ Seeds were sown on agar media containing 0, 30 nM or 10 μM thiamin as described in Mimura et al. (2016). Homozygous mutant *th3* plants were maintained by soil applications of 1 mM thiamin at intervals through flowering.

### Analysis of Thiamin Vitamers

Extraction and quantitative analysis of thiamin vitamers was performed as described in Mimura et al. (2016) and Hasnain et al. (2016). Briefly, whole plants (300 mg) were ground in liquid N_2_ then extracted in 500 ml of 7.2% perchloric acid by 30 min of bath sonication at 0°C. Extracts were incubated on ice for 15 min with periodic vortex mixing then centrifuged to remove cell debris (14,000 g, 10 min, 22°C). Vitamers in the supernatant fluid were converted to thiochrome derivatives and resolved by HPLC with fluorometric detection (Fraccascia et al., 2007; Hasnain et al., 2016).

### RNA-seq Analysis

A multiplex RNA-seq library was constructed from three biological replicate RNA samples each from Landsberg wild-type and *th3* mutant plants grown on media containing 30 nM thiamin as described in Mimura et al. (2016). The library was sequenced in a single direction on a Hiseq500 instrument (Illumina). RNA-seq data were analyzed using TOPHAT (Trapnell et al., 2012) and gene expression quantified using CUFFDIFF (Trapnell et al., 2013). Differentially regulated genes were annotated using the GO-Term Enrichment tool at Arabidopsis.org. Analysis details are summarized in Supplementary Tables S1–S5.

### Analysis of SNPs

The RNA-seq reads were analyzed for *th3* linked single-nucleotide polymorphisms (SNPs) using a kmer-counting approach. In brief, a database of 22-mer oligonucleotide SNP variants was constructed from the set of predicted full-length cDNA sequences for all genes on chromosome 4 using JELLYFISH (Marçais and Kingsford, 2011). In the kmer database, each 22-mer in the wild-type cDNA was represented by four variants including the possible single base substitutions in the first position. Jellyfish was then used to count the frequency of each 22-mer in the RNA-seq data for each replicate. SNP variants were detected by a shift of counts from wild-type kmer to one of the other three variants at that position. SNPs relative to Ler-0 that were present in the mutant replicates but not detected in wild-type RNA-seq reads were selected for further analysis. This method allowed rapid detection of SNPs and quantitative comparison of kmer counts from isogenic wild type and mutant replicates, provided that mutations were sparsely distributed in the coding sequences (i.e., a SNP density well below 1 per 22 bases) – a reasonable expectation for the ems mutagenesis used to generate *th3*.

### Protein Structure Modeling Based on Homology

Structural models the wild-type and *th3* mutant DXS1 proteins were constructed by MODELER (Webb and Sali, 2016) using the *Deinococcus radiodurans* structure as a template (PDB:6OUW; Chen et al., 2019). Fifty replicate models were constructed for each protein, and the objective function score was used to select the best structure (Webb and Sali, 2016). Molecular graphics images were created using the UCSF Chimera^2^ (Pettersen et al., 2004).

## Results

In a search for new genetic leads to identities of genes encoding orphan reactions in the thiamin biosynthesis pathway in plants (Gerdes et al., 2012), we analyzed RNA-seq data to identify candidate genes for the classical thiamin-requiring Arabidopsis mutant *th3*. Using a similar approach, we recently showed that *Th2* encodes thiamin monophosphate (ThMP) phosphatase, filling in a key gap in the thiamin biosynthetic pathway (Mimura et al., 2016). Like *th2*, the *th3* locus is identified by single mutant allele (Koornneef, 1981), which has thus far evaded molecular analysis.

### The *th3* Mutant Is Rescued on Media Supplemented With Thiamin

**Figure 1:**
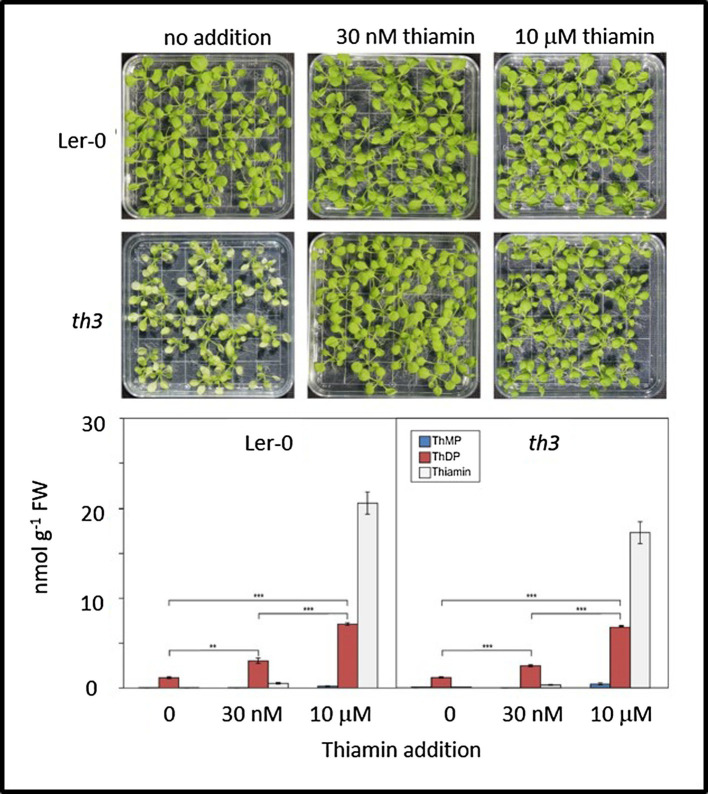
Mutant *th3* seedlings are rescued by thiamin but exhibit no discernible defects in thiamin biosynthesis. Top: phenotypes of wild type (Ler-0) and th3 seedlings grown on MS agar medium containing 0, 30 nM or 10 μM thiamin. Bottom: vitamer profiles of Ler-0 wild type and *th3* seedlings grown on 0, 30 nM or 10 μM thiamin. ThMP, ThDP, and free thiamin were quantified as described in Mimura et al. (2016). Brackets indicate *t*-test comparisons: ^**^p < 0.01 and ^***^p < 0.001. Error bars indicate standard error of the mean for three replicates.

Consistent with Koornneef (1981) characterization of the *th3* mutant, when grown in the absence of supplemental thiamin *th3* mutant plants formed pale-green leaves and grew slowly (Figure 1). We confirmed that *th3* plants could be rescued on media containing thiamin concentrations as low as 30 nM resulting in fertile plants with normal leaves.

### *th3* Mutant Plants Have Normal Vitamer Profiles

Analysis of thiamin vitamers in wild type and *th3* plants (Figure 1) showed no discernible differences in thiamin, ThMP and ThDP contents of mutant and wild-type plants grown either with or without supplemental thiamin in the medium. Growth on medium containing 30 nM thiamin resulted in similar increases in ThDP content in mutant and wild type (2.3-fold and 2.6-fold, respectively) compared to plants grown with no exogenous thiamin. In plants grown on 30 nM thiamin, free thiamin increased relative to plants grown on thiamin-free medium, accounting for 14 and 11% of total thiamin in supplemented wild type and mutant plants, respectively. In plants grown on medium containing a high thiamin concentration (10 μM), free thiamin content increased dramatically in both mutant and wild type. While ThDP content was also elevated at least 6-fold in both mutant and wild type, the elevation in ThDP was not proportional to the large increase in free thiamin content. This result is consistent with evidence from THIC and THI4 overexpression studies indicating that thiamin diphosphokinase is a limiting step in conversion of thiamin to ThDP (Dong et al., 2015). Thus, while the possibility of a lesion affecting transport and subcellular localization of specific vitamers was not ruled out by these data, no obvious defects in thiamin biosynthesis and metabolism were detected in the *th3* mutant.

### The *th3* Mutation Is an Allele of Deoxyxylulose 5-Phosphate Synthase 1

**Figure 2:**
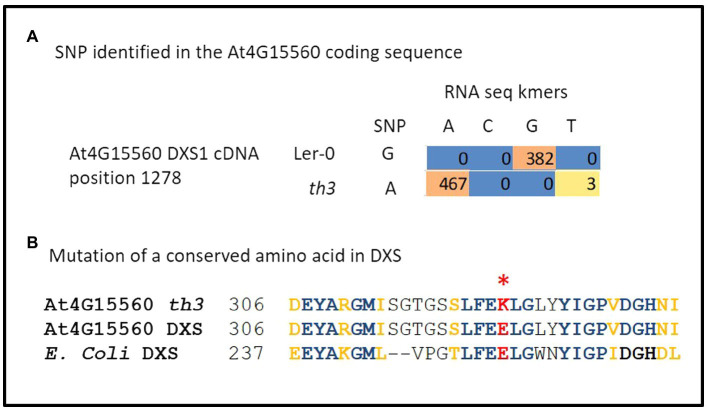
The *th3* genome carries a mutation in *DXS1*. **(A)** Identification of a G to A mutation in At4G15560 encoding DXS1. Coding sequences of all genes located on chromosome 4 were scanned for SNPs using triplicate RNA-seq data from *th3* mutant and wild-type Ler-0 plants grown on 30 nM thiamin. The table shows counts of kmers representing A and G variants in Ler-0 and th3 detected at position 1278 in the full-length cDNA sequence. **(B)** The *th3* associated SNP converts a highly conserved glutamate residue at position 323 in DXS1 sequence to a lysine. The context of the substitution is shown in a partial alignment of Arabidopsis and *E. coli* DXS protein sequences. Identical (blue) and similar (gold) amino acids are highlighted in bold. A non-conservative glutamate to lysine substitution in DXS1-th3 is highlighted in red.

**Figure 3:**
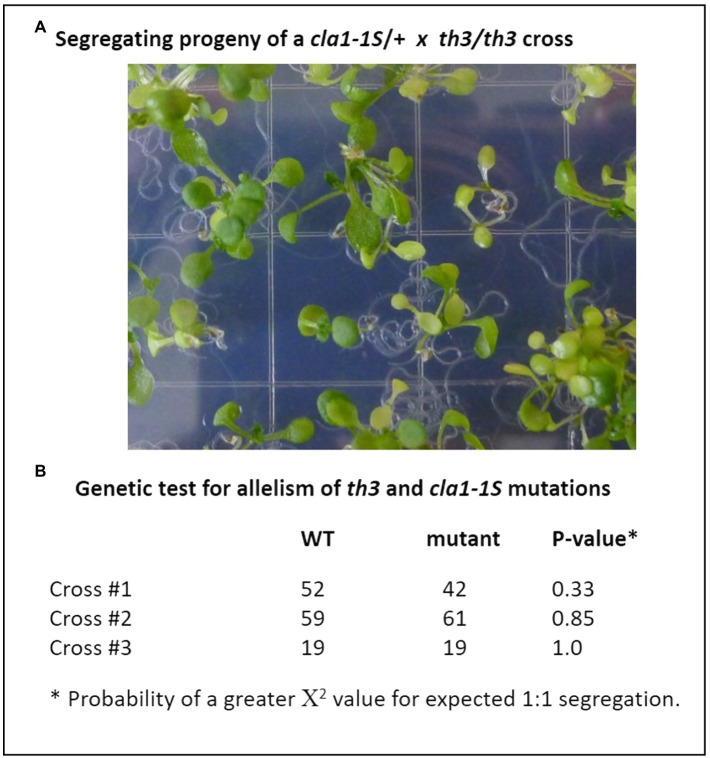
The *th3* mutation is an allele of the *DXS1* (*cla1*) gene. Genetic complementation was tested by crossing *th3* homozygous plants with plants heterozygous for the *cla1-1S* reference allele. **(A)** Segregation of pale-green plants in F1 progeny. Seeds were planted on MS media and seedlings were imaged at 7 days after sowing. **(B)** Expected 1:1 segregation ratios indicative of non-complementation were observed in progeny of three independent crosses. Fit to an expected 1:1 segregation ratio was tested using χ^2^.

In order to identify the mutation responsible for the *th3* phenotype, RNA-seq analysis of triplicate wild type and mutant plant samples was undertaken. Analysis of SNPs in RNA-seq data (Figure 2A) revealed a non-synonymous mutation in the protein coding sequence of AT4G15560, a gene encoding *DXS1*, also known as *Cloroplastos Alterados-1 (CLA1)*. DXS1 is a highly conserved ThDP requiring enzyme that catalyzes the first committed step in the MEP pathway for isoprene biosynthesis in plants and bacteria (Zhao et al., 2013). The location of DXS1 on chromosome 4 is consistent with the classical genetic map position of *th3* locus (Meinke et al., 2009), making the DXS1 SNP a candidate for the *th3* mutation. Consistent with this hypothesis, the mutation in *th3* causes a non-conservative substitution of a lysine for a highly conserved glutamate at position 323 in the DXS1 protein sequence (Figure 2B). To test the allelism hypothesis, homozygous *th3* plants were crossed to plants heterozygous for the *cla1-1S* reference allele. As shown in Figure 3, the F1 seed segregated 1:1 for the characteristic pale-green *th3* phenotype confirming that the *th3* mutant is allelic to the *cla1-1S* reference mutant.

### The *th3* Mutation Alters Charge Distribution at the Interface of DXS1 Subunits

**Figure 4:**
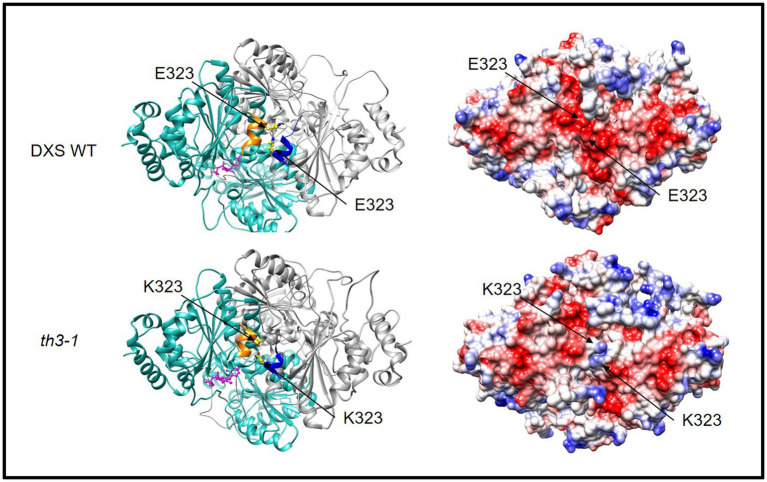
The *th3* E323K mutation occurs at interface of DXS subunits. Glutamate 323 occurs in a short α-helix (gold and blue) that aligns at the interface of the DXS homodimer (ribbon formatted subunits colored light blue and gray, respectively). The lysine substitution alters the electrostatic surface charge of the DXS protein (right). Positive surface charge is colored blue, and negative surface charge is red. Structural models of WT and mutant protein were constructed by homology to DXS of *D. radiodurans* (PDB:6ouw, Chen et al., 2019) using MODELER (Webb and Sali, 2016). Disordered segments adjacent to the conserved α-helix were not included in the model. Graphics were made using the UCSF Chimera (Pettersen et al., 2004).

As shown in Figure 2B, the E323K substitution in the *th3* mutant occurs in a conserved motif (FEELG) that in bacterial DXS enzymes of known structure forms a short α-helix located at the interface between subunits of the DXS homodimer (Chen et al., 2019). To explore implications of the E323K mutation, molecular models of the wild type and *th3* mutant Arabidopsis DXS1 protein were constructed based on homology to the *D. radiodurans* enzyme (PDB:6OUW, Chen et al., 2019) using MODELER (Webb and Sali, 2016). As shown in Figure 4, the face-to-face homodimer configuration places the E323 residues of each wild-type subunit in close proximity at the subunit interface. While the E to K substitution has no predicted effect on the helix structure, the mutation substantially alters the distribution of surface charge on the protein by introducing positive charge at the normally negatively charged subunit interface (Figure 4, right panel). Interestingly, the FEELG α-helix motif is immediately adjacent to a segment (amino acids 198–243) that is not resolved in crystal structures of *E. coli* and *D. radiodurans* DXS proteins due to having a disordered structure. The functional significance of this apparently conserved structural feature is unknown.

### Activation of a Biotic Stress Response in *th3* Plants

**Figure 5:**
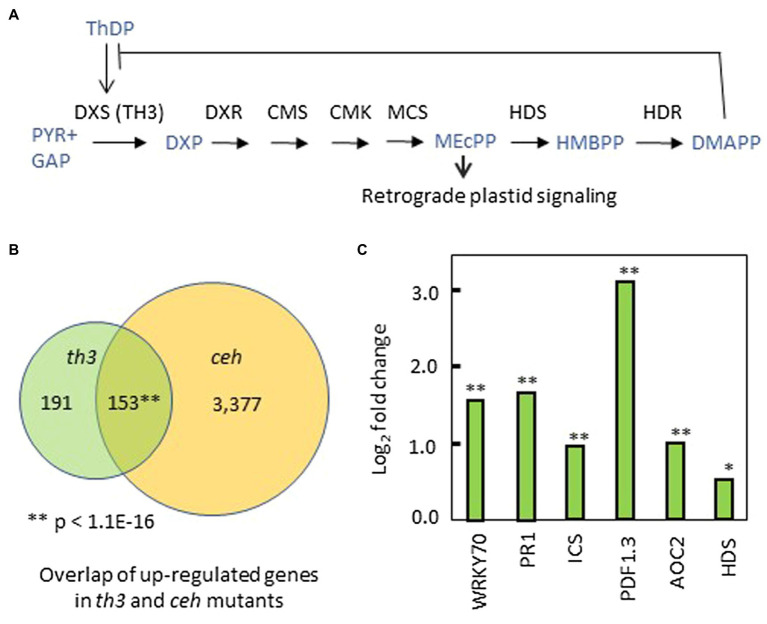
Perturbation of the MEP pathway and plastid signaling by *th3*. **(A)** Mechanisms of regulation in the MEP pathway include DMAPP inhibition of ThDP binding to DXS1 (Banerjee et al., 2013) and retrograde signaling by the MEcPP intermediate (Xiao et al., 2012; Benn et al., 2016). **(B)** Venn diagram showing overlap of genes upregulated in *th3* and *ceh* mutants (Bjornson et al., 2017) above a cutoff of 1.5-fold change (log_2_ fold change > 0.587) in both datasets. ^**^Value of *p* for gene enrichment based a binomial statistic. **(C)** Genes upregulated in thiamin rescued *th3* plants (Supplementary Table S1) include a suite of biotic stress-related genes also induced by *HDS* mutations that accumulate MEcPP (Bjornson et al., 2017). Fold changes in exemplar biotic stress-response genes are shown. In addition, *HDS* is the sole MEP pathway gene differentially affected by *th3*. DXS1, deoxyxylulose-phosphate synthase; DXR, DXP reductoisomerase; CMS, 2-C-methyl-D-erythritol 4-phosphate cytidylyltransferase; CMK, 4-diphosphocytidyl-2-C-methyl-D-erythritol kinase; MCS, 2-C-methyl-D-erythritol 2,4-cyclodiphosphate synthase (MEcPP); HDS, (E)-4-Hydroxy-3-methyl-but-2-enyl pyrophosphate (HMBPP) synthase; HDR, HMB-PP reductase; and DMAPP, Dimethylallyl pyrophosphate. Significant at ^*^p < 0.05; significant at ^**^p < 0.01.

The centrally important MEP pathway for isoprene biosynthesis in chloroplasts is highly regulated in plants (Figure 5A). Known mechanisms include diurnal regulation (Pokhilko et al., 2015), retrograde signaling mediated by the pathway intermediate MEcPP (Xiao et al., 2012; Lemos et al., 2016; Bjornson et al., 2017), and feedback control of DXS1 activity *via* competitive inhibition of ThDP binding by DMAPP (Banerjee et al., 2013). Although *th3* plants rescued on media containing 30 nM thiamin have a normal morphological phenotype, analysis of the replicated RNA-seq data revealed gene expression changes that overlap substantially with patterns conditioned by mutant alleles of HDS that accumulate MEcPP (Xiao et al., 2012; Lemos et al., 2016; Bjornson et al., 2017).

Of 344 genes upregulated in *th3* relative to wild type, 153 genes (Figure 5B, Supplementary Table S1) are also upregulated by the *ceh* mutation of *HDS* (Bjornson et al., 2017) using a threshold of 1.5-fold change for both datasets. The shared suite of upregulated genes is strongly enriched for GO terms associated with biotic stress, pathogen defense responses, and salicylic acid signaling (Supplementary Table S2). Key exemplars of this response are shown in Figure 5C. Interestingly, *HDS* is the sole MEP pathway gene differentially regulated in *th3* plants (Figure 5C). The overlap with *hds (ceh)* affected gene expression is unexpected because effects of *HDS* mutations are attributed to retrograde signaling by MEP intermediate MEcPP that accumulates in *hds* loss of function mutants (Xiao et al., 2012; González-Cabanelas et al., 2015; Benn et al., 2016) and DXS1 over-expressors (Wright et al., 2014). By contrast, attenuation of DXS1 activity causes a reduction in the MEcPP pool size (Wright et al., 2014).

A set of 552 genes downregulated in *th3* (>1.5-fold change, Supplementary Table S3) were enriched for functions associated with abiotic stress responses including water and oxidative stresses (Supplementary Table S2). Of these, 73 genes were also downregulated in *ceh* (Supplementary Table S3). This shared set of genes showed moderate enrichment for cell wall biosynthetic functions (GO:0071554, *p* = 4.07E-03).

## Discussion

Our results show that the classical thiamin-requiring *th3* mutant is an allele of *DXS1* – a key regulated enzyme in the MEP pathway. As expected for a mutation affecting a single thiamin-requiring enzyme, *th3* has no discernible effect on thiamin metabolism overall. We suggest that the *th3* mutation reduces affinity of DXS1 for ThDP cofactor. The physiological implications of this perturbation are likely ramified due to the central importance of the highly regulated MEP pathway in metabolism and signaling.

### Thiamin Responsive Mutations in ThDP Requiring Enzymes

The hypothesis that the *th3* mutation reduces affinity of DXS1 for ThDP is consistent with thiamin responsive mutants studied in other organisms. Notably, there is ample precedence in humans for mutations in thiamin-requiring enzymes having phenotypes that can be rescued by supplemental thiamin (reviewed in Ames et al., 2002). Thiamin responsive mutations that increase the *K*_m_ of human branched chain α-ketoacid dehydrogenase for ThDP are not restricted to the ThDP-binding E1 subunit, highlighting the importance of subunit interactions in determining cofactor affinity (Fisher et al., 1991). Interestingly, structural analysis indicates that the *th3* mutation replaces a highly conserved glutamate (E323) located in a short α-helix at the interface between subunits of the DXS1 homodimer with a positively charged lysine. While it is unknown whether the helix formed by the conserved FEELG motif has a specific role in ThDP binding and assembly of holoenzyme, it is plausible that the change in surface charge alone would affect interaction of the enzyme with the negatively charged ThDP molecule. Whatever the case, our results for DXS1-th3 illustrate that the physiological effects of single-residue changes far from an enzyme’s active site can be as consequential as more easily rationalized changes in the active-site region (Dalby, 2011).

### Sensitivity of DXS1-th3 to *in vivo* Changes in ThDP Concentration

Because the total ThDP content is normally comparable to the abundance of thiamin-dependent enzymes in leaf cells (Hanson et al., 2016; Joshi et al., 2019), the concentration of free ThDP is most likely below the *K*_m_ of DXS1 for ThDP (Banerjee et al., 2013). This suggests that DXS1 is not fully activated under normal conditions rendering the enzyme sensitive to small changes in free ThDP concentration. Indeed, limiting ThDP likely enhances sensitivity of DXS1 to feedback regulation by competitive inhibition of ThDP binding by MEP pathway product, DMAPP (Banerjee et al., 2013; Sharkey et al., 2015).

Activity of the mutant DXS1-th3 enzyme is evidently restored sufficiently by the ~2.5-fold elevation of *in vivo* ThDP content conditioned by supplementation with 30 nM thiamin. However, the observed fold elevation in total ThDP content most likely understates the impact of the 30 nM thiamin supplement on the concentration of free ThDP in leaf cells. If, for example, 90% of the ThDP pool in un-supplemented control plants is bound to enzymes (leaving 10% free), then most of the excess ThDP contained in supplemented plants will be in the unbound fraction. In that instance, a 2.5-fold increase in total ThDP would correspond to a greater than 15-fold increase in free ThDP [(2.5 − 0.9)/0.1 = 16]. Hence, even without taking compartmentalization into account, order-of-magnitude scale changes in free ThDP concentration in plants supplemented with 30 nM thiamin are plausible.

### Perturbation of MEP Pathway Signaling by *th3*

The induction of a biotic stress response associated with MEcPP signaling suggests that the *th3* mutation is a novel genetic perturbation of the MEP pathway. On the one hand, this unexpected biotic stress response in young *th3* plants may reflect complex developmental dependencies of MEcPP signaling. Notably, in young *hds-3* mutant seedlings expression of WRKY70 and PR1 is suppressed relative to wild type in apparent contrast to their induction by MEcPP in older plants (González-Cabanelas et al., 2015). If MEP pathway signaling actively inhibits the stress response in young plants, then attenuation of MEcPP synthesis could result in de-repression of the biotic stress response during an early phase of vegetative development. Alternatively, there are plausible circumstances in which mis-regulation of DXS1 activity in thiamin-supplemented *th3* plants could result in activities that exceed that of wild-type enzyme creating excess flux through the MEP pathway. A key unknown is how *th3* impacts relative sensitivities of DXS1 to changes in *in vivo* concentrations of ThDP and DMAPP. Because DMAPP inhibition of ThDP binding to DXS1 is competitive (Banerjee et al., 2013), implying that cofactor and inhibitor bind to a common site, a DXS1 mutation with a higher *K*_m_ for ThDP will likely also have altered affinity for the DMAPP inhibitor, potentially rendering the enzyme less sensitive to feedback inhibition. In that case, elevated ThDP concentrations that compensate for the reduced cofactor-binding affinity could in principle result in DXS activities that exceed wild-type levels due to insensitivity of the mutant enzyme to feedback inhibition.

### Role of DXS1 in the Thiamin Economy

These findings add to an emerging picture of a highly interconnected thiamin economy in plant cells comprised of enzymes of central metabolism localized in multiple compartments governed by complex dynamics of ThDP synthesis, transport, holoenzyme assembly, and protein turnover. Remarkably, a modest overexpression of transketolase apo-protein in tobacco conditions a pale-green leaf and plant growth phenotype that can be rescued by supplemental thiamin (Khozaei et al., 2015). The effects of excess transketolase apo-protein can be partly attributed to depletion of the pool of ThDP available to other thiamin-requiring enzymes including DXS1. Consistent with that hypothesis, Khozaei et al. showed that the leaf phenotype of transketolase over-expressors is partially rescued on media containing DXS product, deoxyxylulose 5-P. Less intuitively, transketolase overexpression in tobacco also causes reduction in overall thiamin content. Our results showing that thiamin vitamer levels in DXS1-deficient *th3* mutant plants – grown with or without supplemental thiamin (Figure 1) – are normal indicate that reduced flux through the MEP pathway does not detectably alter thiamin metabolism in Arabidopsis. Nevertheless, the complexity of the transketolase overexpression phenotype highlights the apparent sensitivity of the thiamin economy to small perturbations in balance of thiamin-requiring enzymes.

Protein turnover in plastids plays a central role in the thiamin economy. The plastid localized THI4 and THIC proteins that catalyze limiting reactions in the thiazole and pyrimidine branches of the thiamin biosynthetic pathway, respectively, are among the most rapidly degraded proteins in the leaf proteome (Nelson et al., 2014; Li et al., 2017). While DXS1 turnover was not captured in the proteome-wide analyses by Nelson et al. or Li et al., results of Pulido et al. (2016) are consistent with a half-life of less than 5 h in leaves, indicating a turnover rate comparable to those of THI4 and THIC. Thus, three of the most rapidly degraded leaf proteins function in the thiamin economy. The rapid turnover rates of THI4 and THIC thiamin biosynthetic enzymes are likely related to their susceptibility to irreversible inactivation during catalysis. In the most extreme case, THI4 participates in a single-turnover reaction that irreversibly incorporates a sulfur from an active-site cysteine into the thiazole ring of the thiamin precursor (Chatterjee et al., 2011; Joshi et al., 2020). Consequently, one THI4 protein molecule is consumed for every molecule of thiazole synthesized. While THIC is capable of multiple catalytic turnovers, the enzyme is thought to be subject to frequent inactivation during catalysis due to side-reactions involving its radical SAM cofactor (Palmer and Downs, 2013; Hanson et al., 2016). Some thiamin-requiring enzymes may also be susceptible to damage from side-reactions involving reactive intermediates. For example, in addition to its primary activity of catalyzing synthesis of DXS from pyruvate and glyceraldehyde-3-P, bacterial DXS has an oxygenase side-activity that results in decarboxylation of pyruvate forming a reactive peracetate intermediate in the active site (Johnston and Meyers, 2021). Conceivably, if the oxygenase side-reaction occurs in chloroplasts where O_2_ concentrations are high, this reaction could be a significant cause of damage to the enzyme, shortening its functional lifetime. In this context, *th3* provides a tool for probing the relationship between rates of DXS1 turnover (Pulido et al., 2016) and holoenzyme assembly, a poorly understood process implicated in feedback regulation of the MEP pathway (Banerjee et al., 2013). Thus, *th3* is a revealing perturbation of the crucial interface between the thiamin economy and the MEP pathway.

## Data Availability Statement

RNA sequence data used in this study can be accessed at: https://www.ncbi.nlm.nih.gov under Sequence Read Archive project number PRJNA368967.

## Author Contributions

JJ and MM designed and performed experiments and analysed data. MS designed experiments and analysed data. SW performed experiments and bioinformatic analysis of data. JG designed experiments and interpreted results. AH designed experiments, interpreted results and contributed to editing the manuscript. DM designed experiments, analysed data, interpreted results, and principally wrote the manuscript. All authors contributed to the article and approved the submitted version.

## Conflict of Interest

The authors declare that the research was conducted in the absence of any commercial or financial relationships that could be construed as a potential conflict of interest.

## Publisher’s Note

All claims expressed in this article are solely those of the authors and do not necessarily represent those of their affiliated organizations, or those of the publisher, the editors and the reviewers. Any product that may be evaluated in this article, or claim that may be made by its manufacturer, is not guaranteed or endorsed by the publisher.

## Footnotes

^1^ https://abrc.osu.edu/

^2^ https://www.cgl.ucsf.edu/chimera
